# Supplementary material for: Lack of food intake during shift work alters the heart transcriptome and leads to cardiac tissue fibrosis and inflammation in rats
Source: BMC Biol. 2022 Mar 3;20:58. doi: 10.1186/s12915-022-01256-9 (PMC8892784; doi:10.1186/s12915-022-01256-9)
Supplement: Supplementary file 2 — Additional file 2: Figure S1. Effect of shift work on the cycling heart transcriptome using a differential analysis of rhythmicity with dryR (related to Fig. 2). Figure S2. Effect of shift work on the expression of core clock genes in the rat heart (related to Fig. 2). Figure S3. Effect of shift work on the rhythmic expression of genes accounting for enriched KEGG pathways (related to Fig. 2). Figure S4. Analysis of differential gene expression between C, W, and WRF rats (related to Fig. 3). Figure S5. Motif analysis at DNase I hypersensitive sites located within genes rhythmically expressed in the heart of C, W, and WRF rats (related to Fig. 4). Figure S6. KEGG pathway analysis on modules M0 to M37 (related to Fig. 5). Figure S7. Effect of shift work on the expression of genes misregulated by dilated cardiomyopathy (related to Fig. 5). Figure S8. Quantification of picrosirius red staining in the heart (related to Fig. 6). [file 12915_2022_1256_MOESM2_ESM.pdf]

Additional File 2: Supplementary Figures

Lack of food intake during shift work alters the heart transcriptome and leads to cardiac fibrosis and inflammation in rats

Trott AJ, Greenwell BJ, Karhadkar TR, Guerrero-Vargas NN, Escobar C, Buijs RM, Menet JS

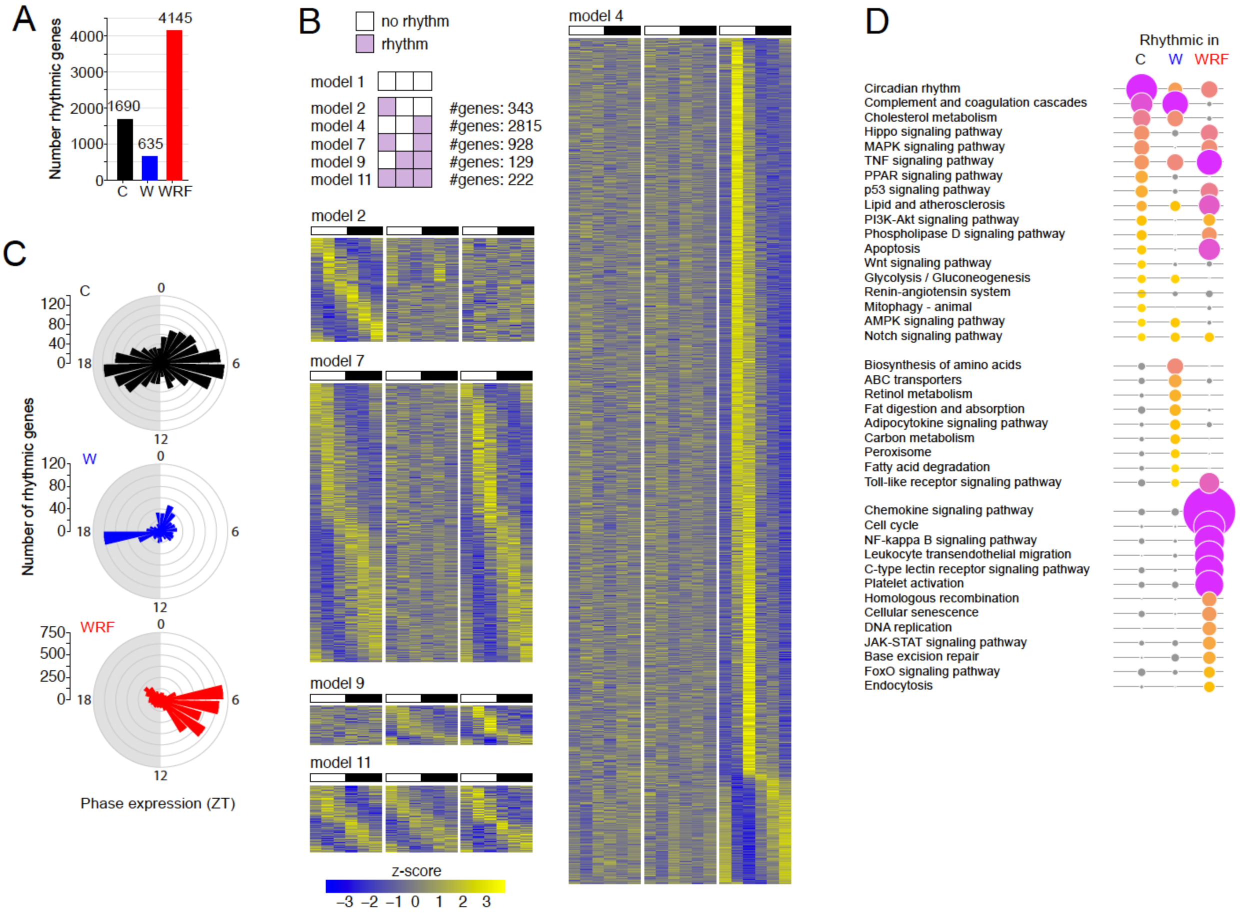

Fig. S1: Effect of shift work on the cycling heart transcriptome using a differential analysis of rhythmicity with dryR

Rhythmic gene expression in the heart of C, W, and WRF rats was analyzed in parallel to the pipeline outlined in Fig. 1 with dryR (Differential Rhythmicity Analysis in R, [23]).

A. Total number of rhythmically expressed genes for each group: control (C, black), shift worker (W, blue), or shift worker subjected to restricted feeding during work (WRF, red).

B. Heatmap visualization of rhythmic gene expression. dryR-generated models containing more than 125 genes are shown (See Table S2 for details). Heatmaps were generated automatically by dryR, and the signal for each timepoint corresponds to the average of 3 individual samples

C. Rose plot representation of the phase of rhythmic gene expression in C, W, and WRF rat groups.

D. KEGG pathway enrichment for genes being rhythmically expressed in C, W, and WRF rat heart. Pathways that are not enriched in certain groups ($p > 0.05$) are displayed in grey. Diameter of each circle is also proportional to the enrichment q-value, with higher diameter for lower q-values.

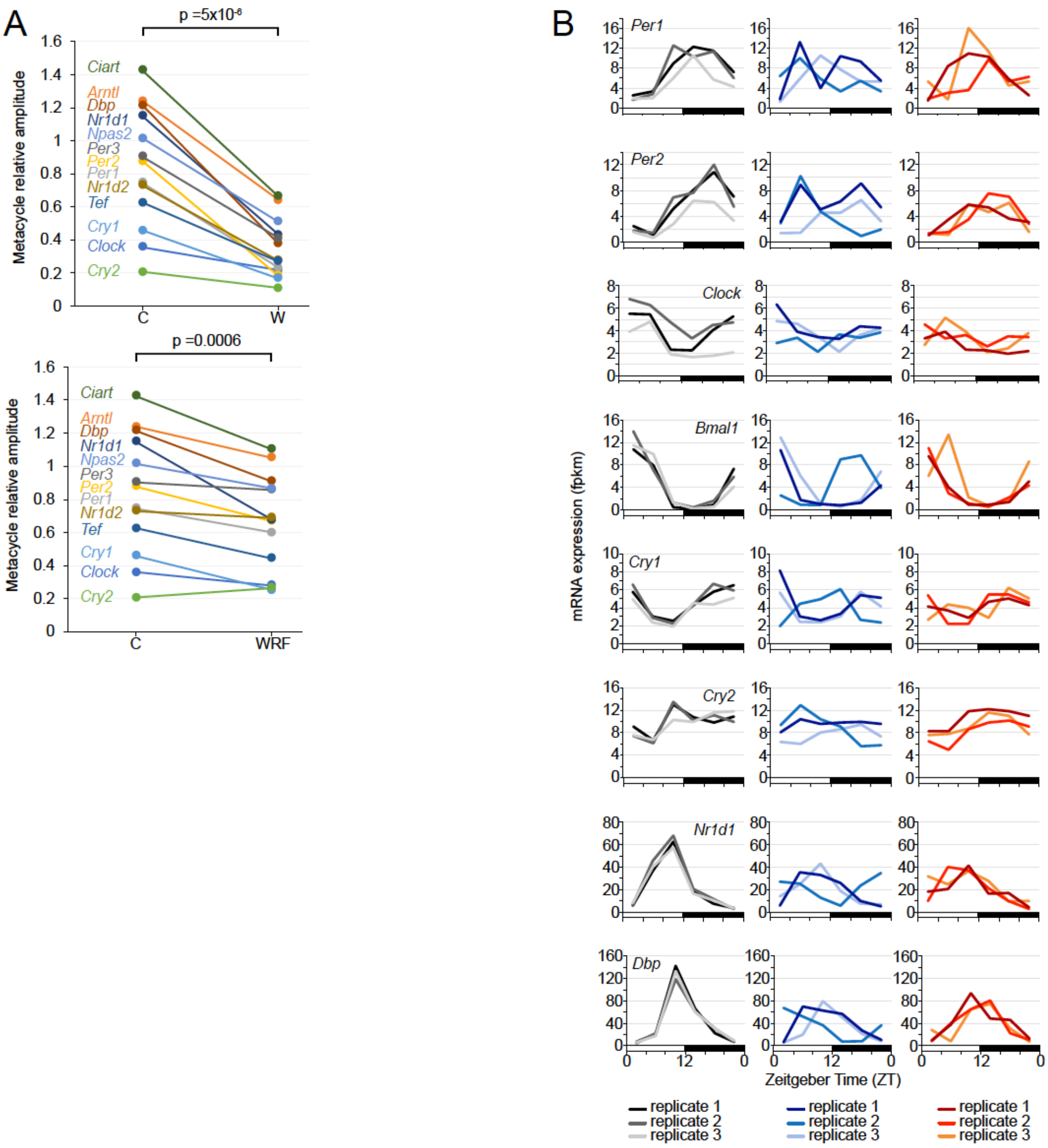

Fig. S2: Effect of shift work on the expression of core clock genes in the rat heart
A. Difference in amplitude of rhythmic expression for 13 core clock genes in the heart of C, W, and WRF rats. The relative amplitude was calculated using Metacycle, and differences between groups was assayed using a two-tailed paired student t-test.
B. Expression of eight core clock genes in the heart of C, W, and WRF rats. The expression is displayed for each of the three independent replicate rhythms for each group. Clock gene peak expression in replicate 2 of W rats is in antiphase to all other replicates.

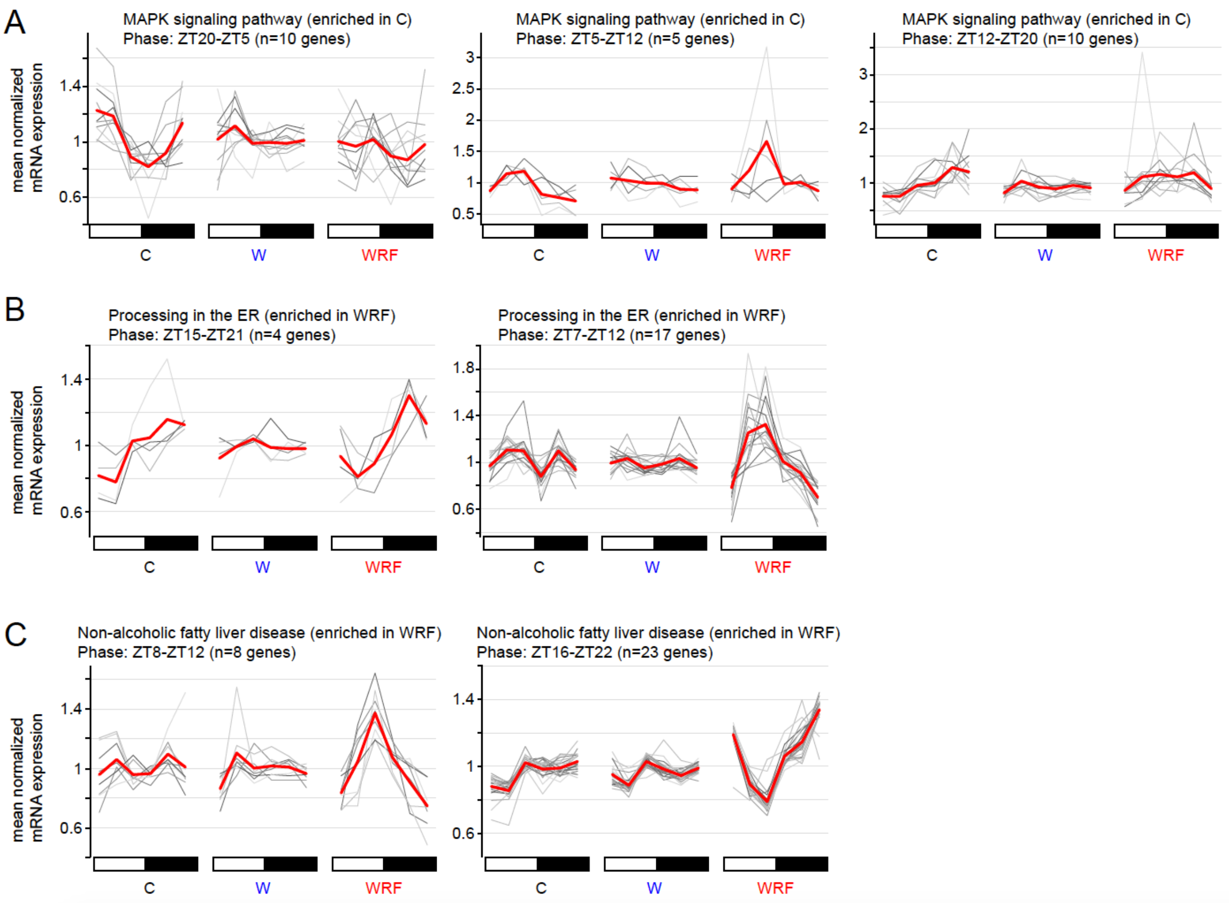

Fig. S3: Effect of shift work on the rhythmic expression of genes accounting for enriched KEGG pathways
Expression profile of genes accounting for the enrichment of three KEGG pathways: MAPK signaling pathway, enriched in C rats; Processing in the ER, enriched in WRF rats; and Non-alcoholic fatty liver disease, enriched in WRF rats. The expression of individual genes is displayed in grey, and the averaged expression in red. For each enriched pathway, gene expression is parsed based on the phase of rhythmic gene expression.

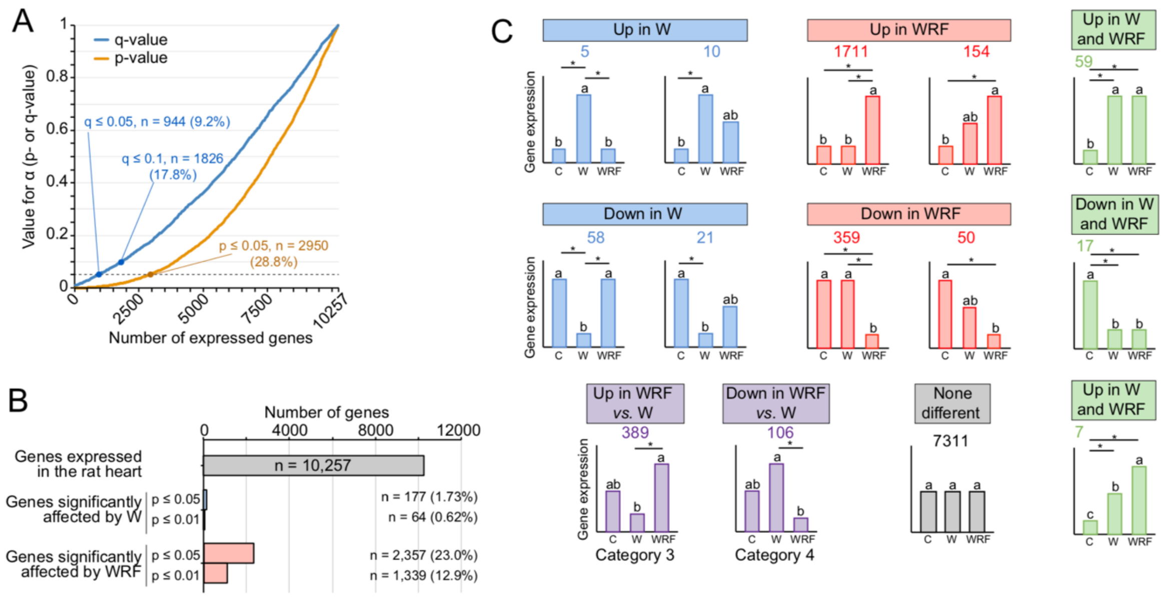

Fig. S4: Analysis of differential gene expression between C, W, and WRF rats
Analysis of differential gene expression between C, W, and WRF rat hearts was conducted using a Kruskal-Wallis test, and returned a p-value for each of the 10,257 genes considered in our analysis. To control for multiple comparisons and set our false-discovery rate at 0.05, we performed a Benjamini-Hochberg correction on each p-value with alpha set at 0.05 (q-value ≤ 0.05). While this stringent analysis allowed us to infer that we have less than 5% of false positives in our list of 944 genes being misregulated by shift work (944 genes with a q-value ≤ 0.05), it is likely that many other genes are affected by shift work since 2,950 of them have a p-value ≤ 0.05.
A. Distribution of p-values and q-values of the Kruskal-Wallis test performed between the C, W, and WRF rats for the 10,257 expressed genes in the rat heart.
B. Number of differentially expressed genes between C, W, and WRF groups based on the p-value threshold (p < 0.01 or p < 0.05)
C. Description of the significant effects between groups after Mann-Whitney U test post hoc analysis when considering a p-value threshold of 0.05 for the Kruskal-Wallis test. Categories of statistically significant differential gene expression are illustrated by bar graphs with the total number of genes written above for each category. Groups with different letters are significantly different.

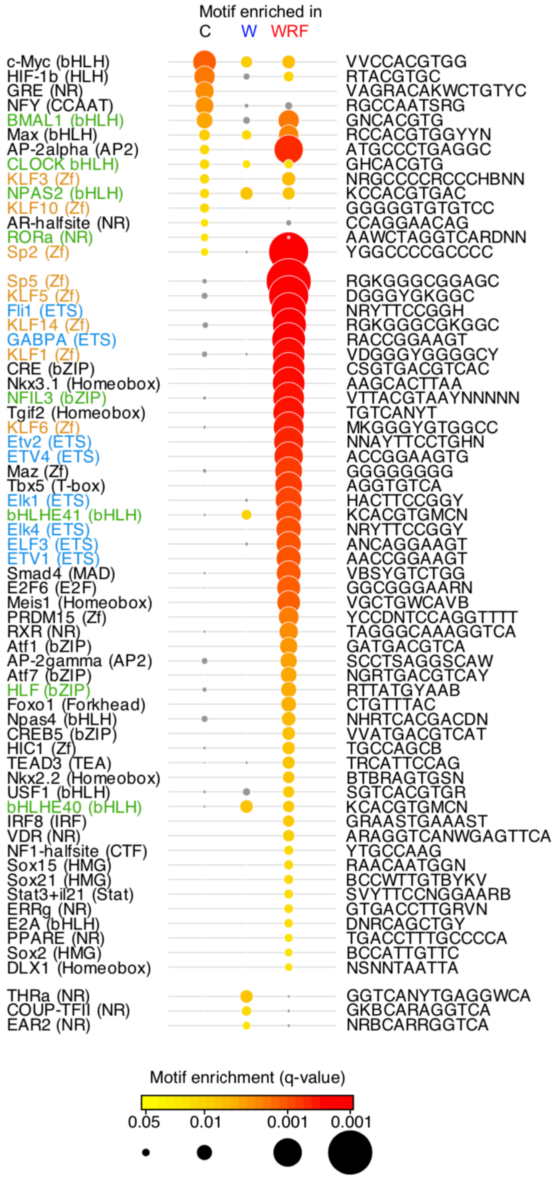

Fig. S5: Motif analysis at DNase I hypersensitive sites located within genes rhythmically expressed in the heart of C, W, and WRF rats
Motifs with a q-value < 0.05 are colored from yellow to red with red being the most significant. Lack of enrichment for a motif (p > 0.05) is displayed in grey. Diameter of each circle is also proportional to the enrichment q-value, with higher diameter for lower q-values. Circadian clock transcription factors are labelled in green, while transcription factors of the ETS family are labeled in blue and those of the SP/KLF family are labeled is orange. The full motif analysis is available as Additional File 1: Table S8.

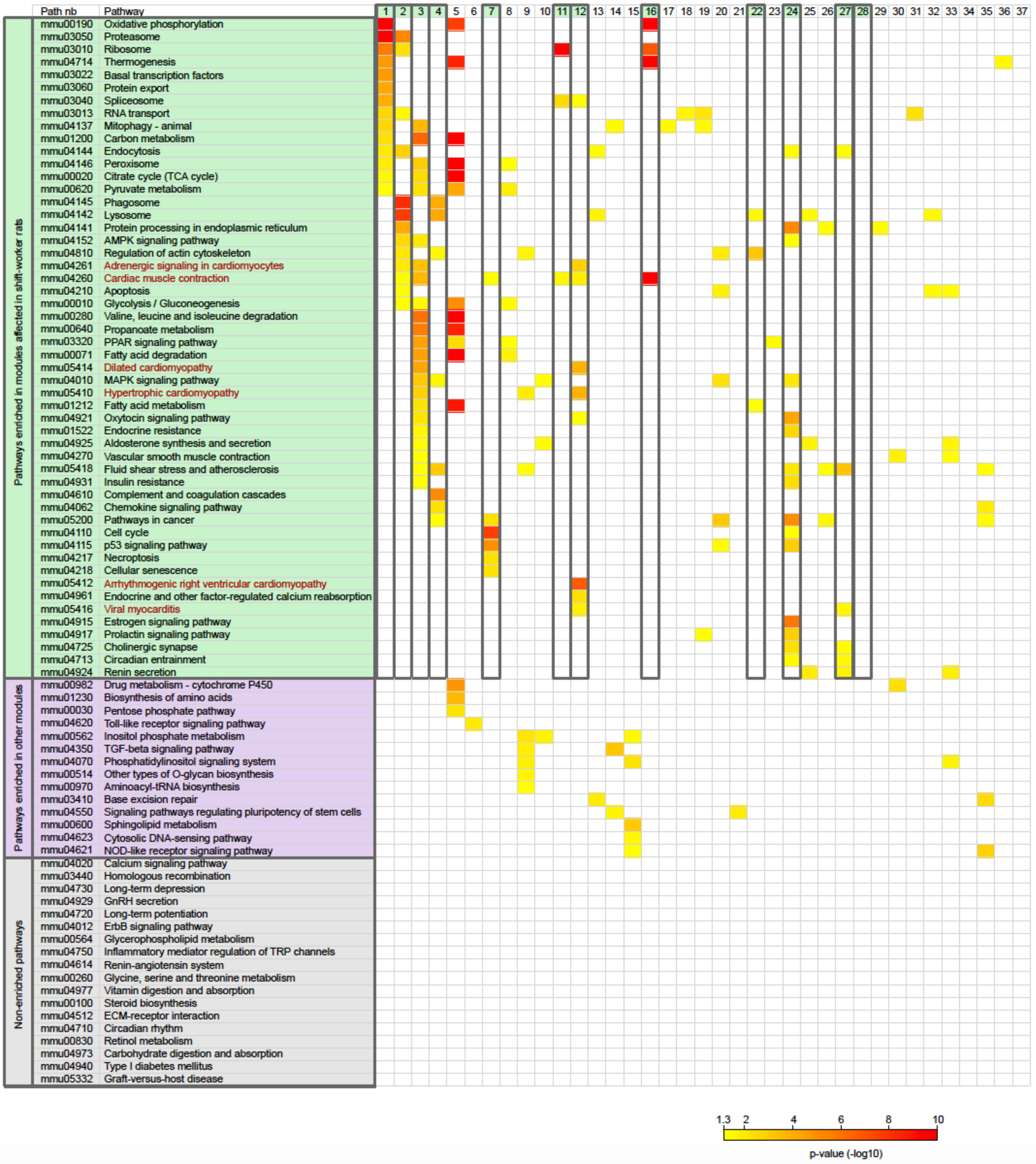

Fig. S6: KEGG pathway analysis on modules M0 to M37
Modules from Nomura et al., 2018 correspond to distinct sets of genes being misregulated by cardiac hypertrophy in the mouse heart [25]. Genes from each module were retrieved and cross-referenced with our list of 10,257 genes expressed in the rat heart. Genes expressed in the rat heart and categorized within each module were used for a KEGG pathway analysis using the function kegga of the R package Limma. The full list of KEGG pathways is provided in Additional File 1: Table S8. Relevant KEGG pathways were selected and their enrichment in modules M1 to M37 are displayed using color-coding. No enrichment is displayed in white.

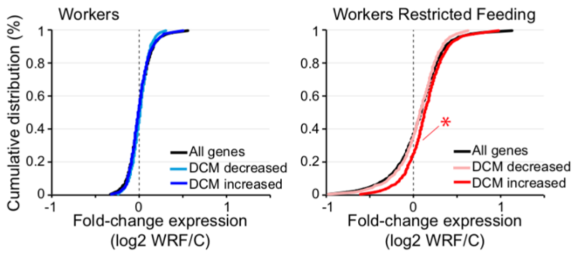

Fig. S7: Effect of shift work on the expression of genes misregulated by dilated cardiomyopathy
Cumulative distribution of WRF/control or W/control expression ratio for all genes (black) or for genes described as either downregulated or upregulated by dilated cardiomyopathy in the human heart. The list of down/up-regulated genes was retrieved from Sweet et al., 2018 [38]. Distribution significant different from that of all genes is illustrated by an asterisk (Kolmogorov-Smirnov test, $p < 0.05$).

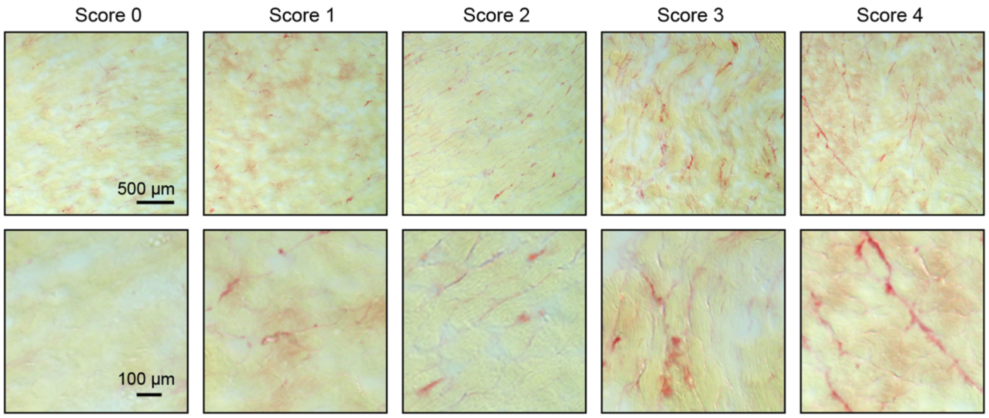

Fig. S8: Quantification of picrosirius red staining in the heart
Representative examples of picrosirius red staining in 10 µm sections of rat heart. A score of 0-4 was given for collagen deposition. Yellow =viable myocardium, and red = collagen.
